# Disseminated Enteroviral Infection Associated with Obinutuzumab

**DOI:** 10.3201/eid2109.150104

**Published:** 2015-09

**Authors:** Claire Dendle, Michael Gilbertson, Tony M. Korman, Vera Golder, Eric Morand, Stephen Opat

**Affiliations:** Monash Health, Clayton, Victoria, Australia (C. Dendle, M. Gilbertson, T.M. Korman, V. Golder, E. Morand, S. Opat);; Monash University, Clayton (C. Dendle, T.M. Korman, V. Golder, E. Morand, S. Opat)

**Keywords:** obinutuzumab, CD 20 monoclonal antibody, enterovirus, dermatomyositis, rituximab, viruses

## Abstract

Two cases of disseminated enteroviral infection occurred in patients who received the CD20 monoclonal antibody obinutuzumab. Clinical features included hepatitis, edema, and a dermatomyositis-like syndrome. These manifestations may be unfamiliar to clinicians and are possibly responsive to intravenous immunoglobulin. Clinicians should remain vigilant for enteroviral infections in patients receiving obinutuzumab.

Viral, fungal, and bacterial infections (*1**,**2*) and a recent case of enteroviral meningoencephalitis (*3*) associated with obinutuzumab use have been described. Early recognition is critical because the infection can be effectively treated with intravenous immunoglobulin (IVIg).We report 2 cases of disseminated enteroviral infections in patients in Australia treated for lymphoma with the CD20 monoclonal antibody (mAb) obinutuzumab. Clinical features, including hepatitis, edema, and a dermatomyositis-like syndrome, were similar to those mentioned in the original descriptions of disseminated enteroviral infections in children with X-linked agammaglobulinemia (XLA) (*4**,**5*). 

## Case Reports

### Case 1

During summer 2014, a 63-year-old woman with symptomatic high tumor burden follicular lymphoma achieved a complete clinical and radiologic response to induction treatment with 6 cycles of bendamustine and obinutuzumab, then began maintenance therapy with obinutuzumab for 8 weeks. Eleven months after she began taking obinutuzumab, the patient sought treatment for 4 weeks of fatigue, myalgias, muscle tenderness, and leg edema without fever. Peripheral blood lymphocyte count was 0.52 × 10^9^ cells/L (reference range 1–4 × 10^9^ cells/L), and lactate dehydrogenase was 354 IU/L (reference range 100–200 IU/L); serum creatine kinase and inflammatory markers were within reference ranges. Immunoglobulin levels were also within reference ranges: IgG 10.2 g/L, IgM 0.3 g/L, and IgA 1.3 g/L. The patient had moderately impaired liver function and was hypoalbuminemic without evidence of renal protein loss. Magnetic resonance imaging of the thighs showed diffuse inflammatory changes involving subcutaneous tissues, fascia, and musculature (Figure). Results of tests to determine possible causes of muscle pathologic changes were negative; tests included those for autoantibodies, HIV antibodies, thyroid function, and PCR for respiratory viruses (including influenza) and herpesvirus. Bone marrow biopsy results indicated no evidence of lymphoma. Muscle histopathologic findings from a biopsy of the quadriceps showed features of an inflammatory myopathy (interstitial edema, perivascular lymphocytic cuffing, and degenerating fibers) consistent with the features of early dermatomyositis. Reverse transcription PCR of the muscle tissue indicated enterovirus RNA. Reverse transcription PCR also detected enterovirus RNA in plasma, nasopharyngeal, and fecal specimens. Viral protein 1 gene obtained from RNA extracted from muscle was sequenced, and we identified the virus as echovirus 6. When we ceased treatment with obinutuzumab and gave the patient 0.8 g/kg IVIg, her symptoms rapidly improved. Results from a repeat plasma enterovirus PCR 11 days after initiation of IVIg were negative.

**Figure:**
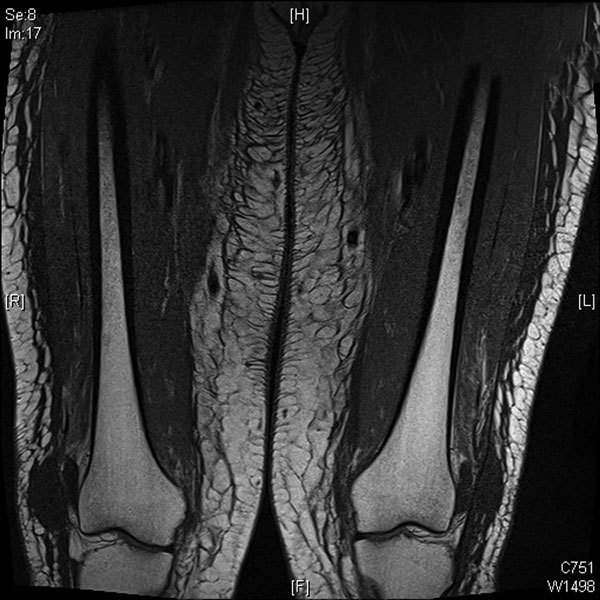
Magnetic resonance image of 63-year-old woman in Australia with disseminated enteroviral infection that manifested after she received obinutuzumab for lymphoma. Image shows patient’s thighs and diffuse inflammatory changes involving subcutaneous tissues, fascia, and musculature.

### Case 2

During summer 2014, a 35-year-old woman with symptomatic follicular lymphoma achieved a complete clinical and radiological response to induction treatment with 6 cycles of bendamustine and obinutuzumab; she subsequently took obinutuzumab for an additional 8 weeks. Twelve months after she began taking obinutuzumab, she sought treatment for fever, headaches, and myalgias. Peripheral blood lymphocyte count was 0.40 × 10^9^ cells/L (*1**,**2**,**4**,**5*). Cerebrospinal fluid was acellular, but we detected enterovirus in cerebrospinal fluid and feces by using PCR. Sequencing of the PCR product was unsuccessful, and we could not identify the enterovirus strain. Immunoglobulin levels were at the lower end of the reference ranges: IgG 7.9 g/L (reference range 7.5–15.6 g/L), IgM 0.6 g/L (reference range 0.5–3.0 g/L), and IgA 1.5 g/L (reference range 0.8–4.5 g/L). Results of liver function tests were initially normal, but liver function deteriorated after 2 weeks. Peak level of bilirubin was 86 μmol/L (reference range 0–20 μmol/L), of alanine amino transferase was 1,419 IU/L (reference range 7–56 UI/L), of alkaline phosphatase was 117 U/L (reference range 30–120 U/L), and of albumin was 28 g/L (reference range 35–45 g/L); international normalized ratio peaked at 2.0 (reference range 0.8–1.2). Results of liver biopsy showed active hepatitis. Results of tests to determine possible causes of hepatitis and encephalitis were negative; the tests included those for autoantibodies, HIV antibodies, thyroid function, and PCR for respiratory viruses (including influenza) and herpesvirus. Bacterial and fungal cultures were negative. Obinutuzumab was ceased, and the patient was treated with 0.8 g/kg IVIg. All clinical and laboratory features rapidly improved.

## Conclusions

Anti-CD20 mABs such as rituximab are now standard of care for treatment of B-cell lymphoma in combination with chemotherapy. The US Food and Drug Administration approved obinutuzumab in September 2013 for use in chronic lymphocytic leukemia, but indications for use probably will expand. Obinutuzumab is glycoengineered to cause more profound and rapid B-cell depletion than rituximab, elicited by subtle differences in the orientation of binding to the CD20 molecule between the 2 drugs (*6*). As a result of these binding differences, compared with rituximab, obinutuzumab has superior induction of apoptosis, natural killer cell activation, and antibody-dependent cytotoxicity but less complement-dependent toxicity (*6*).This mechanism may also explain the differences in susceptibility to, and patterns of, enteroviral infections associated with obinutzumab, resulting in a phenotype similar to XLA (*5*).

Antibodies are the main form of defense against enteroviruses (*7*), and severe, chronic, and disseminated enteroviral infections are generally limited to neonates or patients with profound B-cell deficiencies (XLA or hematopoietic stem cell transplantation). During the 1970s and 1980s, reports described the clinical manifestation of disseminated enterovirus infection in children with XLA (*4**,**5*) and demonstrated that IVIg is an effective therapy for disseminated enterovirus infection (*7**,**8*). Since then, reports of disseminated enteroviral infections have been uncommon. Enteroviral infection has not featured prominently among patients with partial B-cell or immunoglobulin deficiencies, such as patients with chronic variable immunodeficiency (*7*). Immunoglobulin levels of the 2 patients in our study were within reference ranges, but analysis of lymphocyte subsets was not performed. Both patients received the combination of obinutuzumab and bendamustine; it is possible that an association exists between the 2 drugs that results in increased host susceptibility to disseminated enteroviral infection.

The clinical features described in most cases of disseminated enteroviral infections relate to chronic meningoencephalitis (*2**,**5*). However, several reports describe a dermatomyositis-like syndrome with edema and hepatitis that responded to IVIg (*5*); this syndrome is strikingly similar to the cases reported here. Enteroviral infections (coxsackieviruses and echoviruses) also have been implicated in the pathogenesis of myositis (*9*). Enterovirus PCR was positive from the muscle biopsy of the patient in our report, suggesting that the virus had a direct role in pathogenesis of the myositis.

Reports of enteroviral infections associated with rituximab use since its introduction have been rare, in contrast to obibutuzumab, for which a case of enteroviral meningencephalitis has been reported (*2*,*3*). Of the 11 cases of enteroviral infection associated with rituximab use, 8 were meningoencephalitis and 2 were myocarditis (*2*,*10*–*12*). To our knowledge, enteroviral infection has not previously been associated with rituximab use in patients who also had hepatitis, dermatomyositis, and edema, as in the cases we report and those associated with XLA (*5*).

Future studies could define susceptibility to enteroviruses through the effect of obinutuzumab on B-cell and immunoglobulin function and host defense against enteroviral infections. It would be clinically useful to identify biomarkers or clinical predictors of disseminated infection. Future research might also focus on the development of a screening strategy for enteroviral infections followed by prophylactic or preemptive therapy with IVIg.

The clinical manifestation of disseminated enteroviral infections, particularly those similar to dermatomyositis, may be unfamiliar to clinicians caring for adults because most experience of the illness is in children and there have been few reports in recent years. Given the therapeutic response to IVIg in the cases we report, enteroviral infection and the use of IVIg therapy should be considered in patients treated with obinutuzumab who develop atypical clinical features of organ inflammation.
